# Perceived Impact of Wearable Fitness Trackers on Health Behaviours in Saudi Adults

**DOI:** 10.3390/healthcare14010126

**Published:** 2026-01-04

**Authors:** Asma A. Abahussin

**Affiliations:** Department of Biomedical Technology, College of Applied Medical Sciences, King Saud University, Riyadh P.O. Box 10219, Saudi Arabia; asmabahussin@ksu.edu.sa

**Keywords:** wearable fitness trackers, smartwatch, digital health, adverse impact, physical activity, health behaviours, cross-sectional study, Saudi Arabia

## Abstract

**Background/Objectives**: Wearable fitness trackers (WFTs) are growing in popularity as tools to motivate physical activity and support healthier lifestyles. Evidence suggests that they can have both positive and negative effects on user behaviour and well-being. However, little is known about their impact in Saudi settings, considering its unique cultural context. This study aims to investigate the perceived positive and negative impacts of WFTs on adults’ health behaviours and well-being in Saudi Arabia. **Methods**: A cross-sectional survey was conducted among Saudi adults aged 18 years or older who currently use or have previously used WFTs, using an online questionnaire distributed via social media platforms in May 2025. The survey was developed based on evidence from the literature. It included demographic items, five-point Likert-scale questions assessing positive (9 items) and negative (10 items) effects of WFTs, and an open-ended question. Responses were analysed using descriptive statistics, independent samples t-tests, and one-way ANOVA. **Results**: A total of 154 adults participated. The mean composite score for positive effects was 3.26 (SD = 0.73), indicating general agreement on the benefits of WFTs, while the negative effects score was 2.15 (SD = 0.66), showing low endorsement of adverse outcomes. No significant differences appeared between gender (positive: *p* = 0.34; negative: *p* = 0.24) or age groups (positive: *p* = 0.56; negative: *p* = 0.19). However, users of over two months had higher positive scores (M = 3.43, SD = 0.66) than newer or former users (*p* = 0.01). Open responses showed 62% positive experiences; 27% reported stress, guilt, or obsessive monitoring. **Conclusions**: This study provides initial insights into WFT use and perceptions in Saudi Arabia. However, its cross-sectional nature limits the ability to draw causal conclusions. While most users experienced beneficial health outcomes, a significant proportion reported negative psychological experiences. These findings highlight WFT users’ dual experiences and the need for further longitudinal research and diverse recruitment strategies to better understand sustained engagement and psychological effects.

## 1. Introduction

Wearable fitness trackers (WFTs), such as smartwatches and fitness bands, have become increasingly popular tools for encouraging physical activity and promoting healthier lifestyles. These devices provide real-time feedback on health metrics like step count, calories burned, sleep patterns, and heart rate, making them accessible tools for self-monitoring and motivation [1,2,3]. Globally, ownership of wearable fitness trackers has increased by over 9% year-on-year, according to 2023 market estimates [4]. According to global market estimates, more than one billion wearable devices are expected to be in use worldwide by 2028, driven by increasing demand for health-monitoring technologies and personalised wellness [5]. As of 2020, approximately one in five people in the United States use a fitness tracker or smartwatch [6].

In Saudi Arabia, interest in wearable technology is also on the rise. Surveys conducted in 2023 indicated that roughly one-quarter to one-third of Saudi adults, particularly among internet users, own a smartwatch [4]. In fact, Saudi Arabia is among the leading markets in the Middle East for wearables, as it recorded the second-largest wearable device market in the region in 2022 [7]. National digital health initiatives, combined with the observed increasing awareness of lifestyle-related health risks such as obesity and diabetes, further support growth. As a result, WFTs are being adopted not only for fitness tracking but also for broader health management and behavioural change.

Globally, a growing body of research has explored the potential positive effects of WFT use. Documented benefits include increased physical activity, improved motivation, enhanced goal-setting, and greater self-awareness of health behaviours [8,9,10]. The influence of WFTs is grounded in and connected to behavioural change theories such as Self-Determination Theory (SDT) [11]. Several studies have used SDT to explain how WFTs enhance intrinsic motivation by supporting autonomy, competence, and self-regulation through feedback and goal tracking [12,13]. However, emerging studies have also reported potential harms, including anxiety, guilt, frustration, or compulsive monitoring when users fail to meet daily goals [14,15]. These unintended consequences underscore the importance of considering the psychological effects of wearable technologies alongside their physical benefits.

Furthermore, earlier research on WFTs’ behavioural impacts, both positive and negative, has mainly focused on Western populations and may not accurately reflect the sociocultural, behavioural, and regulatory context of Saudi Arabia. For example, Saudi studies show that constructs such as social influence and habit operate differently in Saudi settings, and that cultural values can uniquely shape technology adoption [16,17]. These contextual differences—such as gender norms, health-behaviour patterns, digital health policies, and social network use—emphasise the need for region-specific research. However, limited research exists on the behavioural and emotional impacts of WFTs in Saudi Arabia [17,18,19,20]. Given the increasing use of digital health tools in Saudi Arabia, there is a pressing need to examine both the positive and negative impacts of WFTs on users’ health behaviours, motivations, and overall well-being within this unique cultural context. To address this, this study aims to assess the perceived positive and negative effects of WFTs among adults in Saudi Arabia, drawing on systematically synthesised evidence from existing literature on the perceived impacts of WFTs in other cultural contexts.

## 2. Materials and Methods

A systematic approach was employed to fulfil the aim of the study through two main phases: (1) identifying evidence-based positive and negative impacts of WFTs through a comprehensive review of relevant literature, and (2) developing a questionnaire informed by the identified impacts to investigate their presence and relevance among adults in the Saudi community.

### 2.1. Phase 1: Identifying Evidence of Impacts of WFTs

#### 2.1.1. Search Strategy

On 2 March 2025, a literature search on PubMed was conducted to identify articles studying the impact of WFTs on behaviour and health. PubMed was selected as the sole database due to its comprehensive coverage of health, behavioural, and technology-related literature. The search strategy was developed after testing various combinations of terms to yield the most relevant results. The complete search strategy, including keywords, is presented in Supplementary File S1. The search targeted studies involving adults (≥18 years of age) to align with the target population of this study, and no additional restrictions were applied.

#### 2.1.2. Eligibility Criteria

Articles were included if they met the following criteria: (1) reporting impacts of WFTs on behaviour and health, (2) focused on adults (≥18 years of age), and (3) written in English. Articles that examined the impact of fitness applications (apps) were also included, as WFTs are often associated with apps.

Articles were excluded if they focused on specific populations, such as patients, people with conditions, or the elderly. This is because perceptions and behaviours of such populations may be affected by condition-specific or age-related factors, which limits their generalisability to the wider healthy adult population. Additionally, articles that studied the impact of WFTs in combination with other interventions, such as coaching and training, were excluded.

#### 2.1.3. Article Selection

The article selection process followed two stages. First, articles were screened based on their titles and abstracts. Those who did not meet the eligibility criteria were excluded at this stage. Second, articles identified as potentially relevant during the first stage were read in full and re-examined for eligibility. Articles that failed to meet the inclusion criteria after this assessment were excluded from the study. Two research assistants independently selected the articles across the two stages, and any disagreements were resolved through consensus via discussion with the primary researcher (A.A.A.).

#### 2.1.4. Data Extraction

A data extraction sheet was developed and utilised to gather all relevant information, including the article title, method, identified intervention impact, whether it is considered harmful or positive, the country where the study was conducted, the number of participants, and their gender and age.

### 2.2. Phase 2: Developing an Evidence-Based Questionnaire and Collecting the Data

#### 2.2.1. Questionnaire Structure

A questionnaire was developed based on the comprehensive review of the existing literature conducted in the first stage to explore the perceived positive and negative effects of WFTs in the Saudi community (see Supplementary File S2). Extracted information on positive and negative impacts, behavioural outcomes, and user perceptions from the Phase 1 reviewed articles was synthesised and directly used to generate and refine the questionnaire items. The questionnaire consisted of four main sections:

1. Demographic Information: This section included items on age, gender, and duration and status of wearable fitness tracker use.
2. Positive Effects: This section contained nine statements evaluating the perceived benefits of wearable fitness trackers, including increased physical activity, improved health awareness, and enhanced motivation. Participants rated each item using a five-point Likert scale ranging from 1 (Strongly Disagree) to 5 (Strongly Agree).
3. Negative Effects: This section comprises ten items designed to assess potential drawbacks, including stress from constant monitoring and an obsession with health data. These items were also rated on the same five-point Likert scale.
4. Open-Ended Question: An open-ended item was included to allow participants to elaborate on their personal beliefs, negative perceived consequences, or experiences with WFTs that the closed-ended items may not have captured.

The original version of the questionnaire was developed in Arabic, the primary language of the target population. To ensure clarity, an expert reviewed it, and a series of pilot tests was conducted with individuals from the target group. Feedback from the reviewer and pilots led to several rounds of rewording and refinement of the questionnaire items to enhance simplicity and reduce ambiguity. The questionnaire items were checked for validity, and the internal consistency assessment revealed strong reliability for both primary sections of the questionnaire, with Cronbach’s alphas of 0.89 and 0.87 for the positive effect items (n = 9) and negative effect items (n = 10), respectively. Cronbach’s alpha was employed because the two main sets of items were designed to assess a single construct (positive or negative perceived impacts).

#### 2.2.2. Data Collection and Analysis

The developed questionnaire was used to collect data for the study. Ethical approval for the study was obtained from the Institutional Review Board (IRB) at King Saud University (IRB Project No E-25-10231). The data collection took place in May 2025 through online distribution of the questionnaire link across various social media platforms, including WhatsApp, Twitter (X), and Instagram. The survey link was also distributed through personal networks and community interest groups focused on health, fitness, and technology. The link briefly explained the study, targeting adult Saudis (≥18 years of age) who currently use or have previously used WFTs, to encourage voluntary participation and informed consent. As the survey was self-administered and voluntary, we assumed that only eligible participants would proceed, and eligibility was further confirmed through participants’ responses to questions regarding their age group and WFT use status. The survey design required all items to be completed, and the questions in Sections S1–S3 were structured as compulsory multiple-choice items, which reduced the likelihood of inattentive or incomplete responses. The aim was to recruit about 200 participants, in line with methodological guidelines for exploratory survey research [21]. The final sample included 154 participants, which remains within the acceptable range (100–200) for descriptive studies focused on characterising perceptions rather than testing predefined hypotheses [21].

The collected data were analysed using IBM SPSS Statistics (Version 28). Descriptive statistics were employed to summarise participants’ demographic information and responses to the questionnaire items. Frequencies and percentages were calculated for categorical variables. Means (M) and standard deviations (SD) were used to describe responses to the Likert-scale items related to the positive and negative effects of WFTs. Additionally, composite scores were derived for each theme by averaging the item responses within each category to summarise the perceived impact of WFTs, representing the primary outcomes and ranging from 1 to 5. Higher scores indicate stronger agreement with the positive or negative implications of WFTs. Data were categorised and compared based on gender, age group, and WFT usage duration or status using independent samples t-tests and one-way ANOVA. Open-ended responses were reviewed and categorised into overall themes, positive or negative, for descriptive reporting.

## 3. Results

### 3.1. Phase 1 Results: Systematic Review

#### 3.1.1. Study Selection

**Figure 1:**
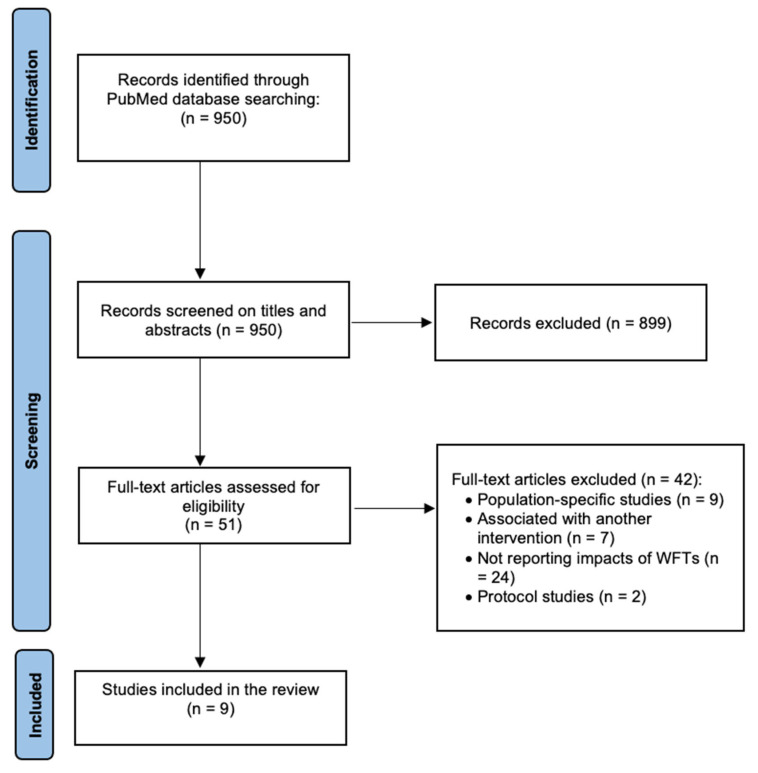
PRISMA diagram.

The Preferred Reporting Items for Systematic Reviews and Meta-Analyses (PRISMA) diagram [22] in Figure 1 illustrates the results of the literature search and selection process. Nine articles met the study’s eligibility criteria and were included in the study.

#### 3.1.2. Study Characteristics

Table 1 presents the extracted data and key characteristics of the nine included studies. The results clearly show that 66.7% (n = 6) of the studies reported a positive impact of WFTs, 22.2% (n = 2) reported both positive and negative effects, and 11.1% (n = 1) reported a mainly negative impact. The reported positive impacts primarily included increased physical activity, improved motivation, and enhanced self-monitoring. In contrast, the negative impacts observed in some studies were related to emotional responses such as anxiety, frustration, or feelings of pressure associated with consistent device use.

### 3.2. Phase 2 Results: Distributed Questionnaire

#### 3.2.1. Descriptive Statistics

A total of 154 adult participants completed the questionnaire assessing perceived positive and negative effects of WFTs. As shown in Table 2, the majority were female (62%), and the largest group was aged 18–29 years (45%), followed by those aged 30–39 years (20%). Regarding tracker usage, 67% of participants were currently using a WFT—53% for more than two months and 14% for less than two months. In contrast, 34% had discontinued use, with 16% having used the device for less than two months before stopping, and 18% for more than two months (Table 2).

#### 3.2.2. Main Questionnaire Results

Participants generally perceived WFTs more positively than negatively, as shown in Table 3. The mean composite score for positive effects was 3.26 (SD = 0.73), reflecting overall agreement with the beneficial impacts of wearable fitness trackers. In contrast, the composite score for adverse effects was 2.15 (SD = 0.66), suggesting relatively low agreement with adverse consequences.

The results of comparing the perceived positive and negative effects of WFTs across gender, age groups, and usage duration, using an independent samples *t*-test and ANOVA, are presented in Table 4. No statistically significant differences were observed between males and females in either positive (*p* = 0.34) or negative (*p* = 0.24) composite scores. Similarly, comparisons across age groups did not reveal significant variations in either side (positive: *p* = 0.56; negative: *p* = 0.19), although participants aged 40–49 reported slightly higher positive and negative scores.

In terms of usage duration, there was a significant difference in positive effect scores across usage duration groups (*p* = 0.01), with participants currently using WFTs for more than two months reporting the highest positive experience. However, negative experience scores did not significantly differ by usage status or duration (*p* = 0.51).

The open-ended responses were categorised through thematic coding. Around 62% of participants shared positive views about using WFTs, noting no adverse experience and often emphasising motivational or health-supportive benefits. Conversely, about 27% of responses revealed negative experiences, with participants describing feelings of stress, pressure to constantly monitor metrics, and guilt when failing to meet daily goals. Some also reported an increasing obsession with their health status and even feelings of depression linked to ongoing self-monitoring. The remaining responses (approximately 11%) were not directly related to health behaviours; some mentioned technical issues or cost-related concerns, while others lacked explicit or meaningful content.

## 4. Discussion

This cross-sectional study aimed to assess the perceived positive and negative effects of WFTs on health behaviours among adults in Saudi Arabia, based on a set of impacts systematically derived from the existing literature. The literature review revealed that almost 67% of the included studies reported a positive effect of WFTs, while the remaining 33% identified either both positive and negative impacts or primarily adverse outcomes. The most reported benefits included increased physical activity [8,9,26], improved motivation [9,10], better weight management [9,15,24], and enhanced self-monitoring [9,10]. In contrast, the adverse effects centred around emotional responses, such as anxiety, frustration, and pressure from consistent device use and metrics tracking [14,15,23] (Table 1).

Our study findings largely reflected the patterns observed in the literature, with participants generally viewing WFTs more positively than negatively. Responses showed a high agreement score of 3.26 for positive effects and a lower score of 2.15 for adverse effects (Table 3). This suggests that concerns about adverse outcomes existed but were less strongly felt in the Saudi context. These findings were further supported by the open-ended responses, in which most participants (62%) expressed positive views about WFTs. At the same time, a notable proportion (27%) described negative experiences such as stress, guilt, obsessive monitoring, and, in some cases, feelings of depression or health-related anxiety. This alignment between the structured and qualitative data reinforces the relevance of previously documented impacts. It highlights both the motivational potential and psychological challenges associated with WFT use in the Saudi context, as well as the need for a balanced consideration of both sides. Studies have confirmed the need to explore the potential factors influencing both sides of the effects, suggesting that the user’s complex characteristics, such as mental health and level of education, play a significant role in this regard [14,27]. As these underlying factors might influence perceptions, future research should include such variables to allow for more thorough analyses.

It is worth noting that a statistically significant difference appeared in positive experience scores across usage duration groups (Table 4, *p* < 0.05), with participants currently using WFTs for more than two months (n = 81, 53%, Table 2) reporting the highest positive experiences (M = 3.43, SD = 0.66). On the other hand, those who had discontinued use after less than two months (n = 24, 16%, Table 2) reported the lowest (M = 2.93, SD = 0.67). These findings suggest that participants who reported fewer positive experiences may be more inclined to discontinue use, as limited perceived benefits can reduce motivation to continue using the device. Conversely, higher perceived benefits are likely to enhance motivation and support sustained use. This interpretation aligns with behavioural perspectives, suggesting that continued engagement is reinforced when individuals experience meaningful benefits and feel competent and autonomous—core components of sustained motivation according to Self-Determination Theory [11]. The findings also indicate that prolonged and consistent engagement with WFTs is associated with greater perceived benefits, consistent with other studies [28,29]. Furthermore, WFTs are unlikely to influence physical activity unless they are worn regularly over an extended period. Various factors influencing long-term engagement with WFTs have been identified in different international contexts [28,29]. Further research is necessary to explore how these factors apply within the Saudi context.

The study found no significant differences in responses between genders or across age groups (Table 4, *p* > 0.05). Gender and age were also found to have no significant impact on examining other aspects of user experience with WFTs [28]. This suggests that the experiences of WFTs are consistent across these demographic groups within the Saudi community, similar to other settings, highlighting again the importance of considering broader factors beyond basic demographics to better understand what drives both positive engagement and negative consequences in diverse populations.

Although there were no significant differences across age groups, the sample was skewed toward younger adults (n = 69, 45%, Table 4). Interestingly, participants aged 40–49 (n = 29, 19%) reported slightly higher positive and negative experience scores than other groups, including younger participants. This contrasts with the usual pattern that younger adults are more engaged with digital technologies and self-tracking. Possibly, individuals 40–49 are more health-conscious and motivated to improve fitness, increasing perceived benefits of wearables. At the same time, their greater health awareness and routines may heighten sensitivity to accuracy concerns or pressure, leading to higher negative perceptions. These findings highlight age-related differences that may not align with typical assumptions about technology use.

## 5. Strengths and Limitations

This study offers a crucial initial insight into the perceived effects of WFTs and user experiences within the Saudi community. This area has been relatively underexplored in existing research. One of the main strengths of this study is its evidence-informed design, where the survey items were developed based on a review of existing literature to capture both the positive and negative effects of WFTs. The inclusion of both structured Likert-scale items and open-ended responses added depth to the findings, allowing for a more comprehensive understanding of user perceptions.

However, the study also has limitations. Its cross-sectional nature limits the ability to draw causal conclusions, and while the sample size was reasonable, it may not fully capture the diversity of the broader Saudi population. Due to time constraints for conducting the study, data collection was limited to one month, which affected the ability to recruit a larger and more diverse sample. With a longer data collection window, a broader participant base could have been achieved. In addition, although the questionnaire was randomly distributed via social media platforms, the final sample was disproportionately female. This gender imbalance may reflect higher engagement among women with health-related surveys or WFT use, which other studies have also observed [23,29]. It also limits the generalisability of the findings as male perspectives, particularly regarding positive impact or negative experiences of WFTs, may be underrepresented. Additionally, using social media as a recruitment platform may have introduced age bias, as older adults are underrepresented. It could also reduce representativeness, since individuals who are active on social media or more engaged with technology may be overrepresented.

Lastly, although the open-ended responses enriched the data, they were relatively brief and lacked depth, which may have constrained the thematic analysis. Given the limitations discussed, the results of this study should be interpreted with caution and regarded as an initial step in examining the perceived impacts of WFTs in the Saudi setting. Future research could address these limitations through longitudinal designs, more diverse and systematic recruitment strategies, and in-depth qualitative interviews to better understand sustained WFT engagement and its psychological impacts.

## 6. Conclusions

Our study provides an initial exploration of the perceived positive and negative effects of WFTs among adults in Saudi Arabia. By building on evidence-based impacts identified in the literature, the study offers a culturally relevant perspective on how WFTs influence health behaviours in a local context. The findings revealed that while most participants reported positive experiences—such as increased physical activity, motivation, and health awareness—a notable proportion also experienced psychological drawbacks, including stress, guilt, and obsessive monitoring. These results were consistent across gender and age groups but varied according to duration of use, with long-term users reporting a higher positive impact. Future research should explore long-term outcomes, recruit more balanced and diverse samples, use a mixed-methods approach, and analyse local factors affecting sustained WFT use to better understand users’ experiences with such devices and develop strategies that reduce negative psychological responses and promote healthier engagement.

## Figures and Tables

**Table 1:** Characteristics of the included studies.

Study	Method	Impact	Type of Impact	Country	Number of Participants	Gender (% Female)	Average Age
[8]	Randomised Controlled Trial	Increase in steps.	positive	United States	n = 265	66.0%	39.9
[15]	Mixed-method	Decrease in BMI and increase in daily step counts.	positive	Australia	n = 55	51.0%	23.6
		Negative feeling and demotivation from the social comparison with fitter people in the app.	negative				
[14]	Mixed-method	(1) guilt formation because of the nature of persuasive models, (2) social isolation as a result of personal regimens around diet and fitness goals, (3) fear of receiving negative responses when targets are not achieved, and (4) feelings of being controlled by the app.	negative	England	n = 117	27.4%	18–25
[23]	Survey	Anxiety or frustration when prevented from wearing their device.	negative	Australia	n = 237	72.0%	33.1
		Positive experience for users with little risk of negative psychological consequences.	positive				
[10]	Systematic review	Behaviour change towards self-improvement identified from 83 articles including: self-monitoring, goal setting, reinforcement, self-awareness, and self-knowledge, increase physical activity and manage weight.	positive	NA	NA	NA	NA
[9]	Randomised pilot study	Increase in physical activity and decreased daily caloric intake. Improvement in self-efficacy, social support and intrinsic motivation.	positive	United States	n = 38	73.6%	21.5
[24]	Systematic review	Improve diet, physical activity and sedentary behaviours.	positive	NA	NA	NA	NA
[25]	Systematic review and meta-analyses	A small-to-moderate positive effect on physical activity measures.	positive	NA	NA	NA	NA
[26]	Feasibility study with pre-post intervention measures	Increase in physical activity.	positive	Australia	130	52.3%	23.7

NA: Not Applicable.

**Table 2:** Participant Demographics (n = 154).

Variable	Category	n (%)
Gender	Female	96 (62%)
	Male	58 (38%)
Age Group (years)	18–29	69 (45%)
	30–39	30 (20%)
	40–49	29 (19%)
	50–59	22 (14%)
	60+	4 (3%)
WFT Usage Duration/Status	Currently using—<2 months	21 (14%)
	Currently using—>2 months	81 (53%)
	No longer using—used <2 months	24 (16%)
	No longer using—used >2 months	28 (18%)

**Table 3:** Perceived positive and negative effects of wearable fitness tracker use (n = 154).

Question No.	Positive Effect Section (M ± SD)	Negative Effect Section (M ± SD)
1	3.63 ± 0.98	2.53 ± 1.10
2	3.82 ± 1.00	1.87 ± 0.93
3	3.75 ± 0.97	2.35 ± 1.02
4	3.16 ± 1.10	2.44 ± 1.05
5	3.23 ± 1.05	2.27 ± 0.98
6	2.67 ± 1.07	2.45 ± 0.97
7	2.83 ± 1.02	1.63 ± 0.87
8	2.46 ± 0.90	1.73 ± 0.86
9	3.78 ± 1.04	1.89 ± 0.89
10		2.36 ± 1.11
Composite Score	3.26 ± 0.73	2.15 ± 0.66

**Table 4:** Comparison of composite positive and negative effect scores by gender, age group, and usage duration.

Group (n)	Positive Effects (M ± SD)	Negative Effects (M ± SD)	*p*-Value (Positive) (95% CI, df) *	*p*-Value (Negative) (95% CI, df) *
Male (n = 58)	3.23 ± 0.87	2.20 ± 0.66	0.34 (−0.20 to 0.32, 94.35)	0.24 (−0.29 to 0.14, 121.94)
Female (n = 96)	3.28 ± 0.64	2.12 ± 0.67		
18–29 (n = 69)	3.23 ± 0.64	2.14 ± 0.68	0.56 (−0.25 to 0.30, 149)	0.19 (−0.22 to 0.20, 149)
30–39 (n = 30)	3.20 ± 0.70	2.05 ± 0.65		
40–49 (n = 29)	3.47 ± 0.78	2.34 ± 0.54		
50–59 (n = 22)	3.17 ± 0.86	2.18 ± 0.73		
60+ (n = 4)	3.19 ± 1.46	1.58 ± 0.53		
Currently using < 2 months (n = 21)	3.11 ± 0.79	2.23 ± 0.73	0.01 (−0.10 to 0.16, 150)	0.51 (−0.27 to 0.32, 150)
Currently using > 2 months (n = 81)	3.43 ± 0.66	2.14 ± 0.70		
No longer using < 2 months (n = 24)	2.93 ± 0.67	2.28 ± 0.56		
No longer using > 2 months (n = 28)	3.16 ± 0.85	2.03 ± 0.58		

* CI: Confidence Interval, df: Degrees of freedom (within groups).

## Data Availability

Data available on reasonable request due to ethical restrictions.

## Supplementary Materials

The following supporting information can be downloaded at: https://www.mdpi.com/article/10.3390/healthcare14010126/s1, File S1: Search strategy; File S2: Questionnaire items.
