# Supplementary material for: Perceived Impact of Wearable Fitness Trackers on Health Behaviours in Saudi Adults
Source: Healthcare (Basel). 2026 Jan 4;14(1):126. doi: 10.3390/healthcare14010126 (PMC12786264; doi:10.3390/healthcare14010126)
Supplement: Supplementary file 1 [file healthcare-14-00126-s001.zip › File S1.pdf]

File S1: Search strategy

Concept	Search number	Query
**Effects**	**1**	Outcome*[Title/Abstract] OR Effect*[Title/Abstract] OR Implication*[Title/Abstract] OR Influence*[Title/Abstract] OR consequence*[Title/Abstract] OR impact*[Title/Abstract]
**Wearable Fitness Trackers**	**2**	"apple watch*"[Title/Abstract] OR tracker*[Title/Abstract] OR "fitness track*"[Title/Abstract] OR Fitbit[Title/Abstract] OR Smartwatch*[Title/Abstract] OR "Fitness wearable*"[Title/Abstract] OR "fitness app*"[Title/Abstract]
**Health and Behaviours**	**3**	behavior*[Title/Abstract] OR behaviour*[Title/Abstract] OR health[Title/Abstract] OR lifestyle[Title/Abstract] OR activit*[Title/Abstract] OR Physical[Title/Abstract] OR diet[Title/Abstract] OR eating[Title/Abstract] OR exercise[Title/Abstract] OR workout[Title/Abstract] OR weight[Title/Abstract] OR Sleep*[Title/Abstract] OR habit*[Title/Abstract] OR Mental[Title/Abstract] OR wellbeing[Title/Abstract] OR psychological[Title/Abstract]
**All concepts**	**4**	((Outcome*[Title/Abstract] OR Effect*[Title/Abstract] OR Implication*[Title/Abstract] OR Influence*[Title/Abstract] OR consequence*[Title/Abstract] OR impact*[Title/Abstract]) AND ("apple watch*"[Title/Abstract] OR tracker*[Title/Abstract] OR "fitness track*"[Title/Abstract] OR Fitbit[Title/Abstract] OR Smartwatch*[Title/Abstract] OR "Fitness wearable*"[Title/Abstract] OR "fitness app*"[Title/Abstract])) AND (behavior*[Title/Abstract] OR behaviour*[Title/Abstract] OR health[Title/Abstract] OR lifestyle[Title/Abstract] OR activit*[Title/Abstract] OR Physical[Title/Abstract] OR diet[Title/Abstract] OR eating[Title/Abstract] OR exercise[Title/Abstract] OR workout[Title/Abstract] OR weight[Title/Abstract] OR Sleep*[Title/Abstract] OR habit*[Title/Abstract] OR Mental[Title/Abstract] OR wellbeing[Title/Abstract] OR psychological[Title/Abstract])
