# Supplementary material for: Perceived Impact of Wearable Fitness Trackers on Health Behaviours in Saudi Adults
Source: Healthcare (Basel). 2026 Jan 4;14(1):126. doi: 10.3390/healthcare14010126 (PMC12786264; doi:10.3390/healthcare14010126)
Supplement: Supplementary file 1 [file healthcare-14-00126-s001.zip › File S2.pdf]

File S2: Questionnaire items

Section S1: Demographic Information

1. Gender
    a. Male
    b. Female
2. Age (years)
    a. 18-29
    b. 30-39
    c. 40-49
    d. 50-59
    e. 60+
3. Duration of Wearable Fitness Tracker Use
    a. Currently using – for less than 2 months
    b. Currently using – for more than 2 months
    c. No longer using – used for less than 2 months
    d. No longer using – used for more than 2 months

Section S2: Positive Effects

1. Using the smartwatch has helped increase my physical activity.
2. The smartwatch makes it easier for me to monitor my daily performance, which motivates me to adopt healthier behaviours and improve my physical fitness.
3. Notifications from the smartwatch encourage me to increase my daily steps and meet my step goals.
4. My physical activity level is higher when I wear the smartwatch compared to when I do not.
5. Using the smartwatch has improved my overall quality of life, including satisfaction, happiness, and both physical and psychological well-being.
6. My sleep patterns have improved since I started using the smartwatch.
7. Using the smartwatch has supported my efforts to lose weight.
8. The smartwatch has helped me reduce food intake and improve my diet, contributing to lower daily calorie consumption.
9. I recommend the use of smartwatches or fitness trackers to improve others' quality of life.

Section S3: Negative Effects

1. I feel frustrated or discouraged when I compare my performance with that of other users (e.g., number of steps taken or calories burned).
2. I tend to engage in binge eating when I do not meet my daily health goals.
3. I experience anxiety or discomfort when I am not wearing my smartwatch.
4. Not meeting daily health goals (e.g., steps taken, sleep hours, or calories burned) increases my stress when using the smartwatch.
5. I feel anxious or upset about receiving negative notifications from the smartwatch when I fail to meet my daily goals.

6. Motivational notifications from the smartwatch sometimes increase my feelings of guilt.
7. I may engage in unhealthy behaviours, such as food restriction or self-induced vomiting, to meet daily goals set by the smartwatch.
8. I may avoid social interactions or decline invitations to strictly follow the health plan and achieve daily goals set by the smartwatch.
9. I feel constrained by the smartwatch, as it requires my activities and diet to align with its predefined daily goals.
10. Using the smartwatch has led me to monitor my performance excessively (e.g., checking health indicators more than twice a day).

Section S4: Open-Ended Question

1. Do smartwatches have any adverse effects on health behaviours? If so, what are they in your opinion?
